# Imatinib Inhibits GH Secretion From Somatotropinomas

**DOI:** 10.3389/fendo.2018.00453

**Published:** 2018-08-27

**Authors:** Prakamya Gupta, Ashutosh Rai, Kanchan Kumar Mukherjee, Naresh Sachdeva, Bishan Das Radotra, Raj Pal Singh Punia, Rakesh Kumar Vashista, Debasish Hota, Anand Srinivasan, Sivashanmugam Dhandapani, Sunil Kumar Gupta, Anil Bhansali, Pinaki Dutta

**Affiliations:** ^1^Department of Neurosurgery, Postgraduate Institute of Medical Education and Research, Chandigarh, India; ^2^Department of Endocrinology, Postgraduate Institute of Medical Education and Research, Chandigarh, India; ^3^Department of Histopathology, Postgraduate Institute of Medical Education and Research, Chandigarh, India; ^4^Department of Histopathology, Government Medical College and Hospital, Chandigarh, India; ^5^Department of Pharmacology, Postgraduate Institute of Medical Education and Research, Chandigarh, India

**Keywords:** growth hormone, imatinib, somatotropinoma, c-kit, VEGF, PDGFR-α/β

## Abstract

**Background:** Imatinib, a tyrosine kinase inhibitor, causes growth failure in children with chronic myeloid leukemia probably by targeting the growth hormone (GH)/insulin like growth factor-1 (IGF-1) axis. We aim to explore the imatinib targets expression in pituitary adenomas and study the effect of imatinib on GH secretion in somatotropinoma cells and GH3 cell line.

**Materials and Methods:** The expression pattern of imatinib's targets (c-kit, VEGF, and PDGFR-α/β) was studied using immunohistochemistry and immunoblotting 157 giant (≥4 cm) pituitary adenomas (121 non-functioning pituitary adenomas, 32 somatotropinomas, and four prolactinomas) and compared to normal pituitary (*n* = 4) obtained at autopsy. The effect imatinib on GH secretion, cell viability, immunohistochemistry, electron microscopy, and apoptosis was studied in primary culture of human somatotropinomas (*n* = 20) and in rat somato-mammotroph GH3 cell-line. A receptor tyrosine kinase array was applied to human samples to identify altered pathways.

**Results:** Somatotropinomas showed significantly higher immunopositivity for c-kit and platelet-derived growth factor receptor-β (PDGFR-β; *P* < 0.009 and *P* < 0.001, respectively), while staining for platelet-derived growth factor receptor-α (PDGFR-α) and vascular endothelial growth factor (VEGF) revealed a weaker expression (*P* < 0.001) compared to normal pituitary. Imatinib inhibited GH secretion from both primary culture (*P* < 0.01) and GH3 cells (*P* < 0.001), while it did not affect cell viability and apoptosis. The receptor tyrosine kinase array showed that imatinib inhibits GH signaling via PDGFR-β pathway.

**Conclusion:** Imatinib inhibits GH secretion in somatotropinoma cells without affecting cell viability and may be used as an adjunct therapy for treating GH secreting pituitary adenomas.

## Introduction

The tyrosine kinase inhibitor (TKI) imatinib is the first-line treatment for chronic myeloid leukemia (CML) and gastrointestinal stromal tumors (GIST). Growth failure or growth retardation has been observed in children treated for breakpoint cluster region-Abelson (BCR-ABL)-positive leukemia, especially if treated before the onset of puberty (1–5). The mechanism of action leading to inhibition of the GH-IGF-1 axis remains undeciphered.

Hypersecretion of growth hormone (GH) causes acromegaly and is invariably due to somatotropinoma. Although the past few decades have seen tremendous improvement in the management of somatotropinomas by the use of somatostatin and dopamine agonists and GH receptor antagonists. The limitations imposed by their cost and efficacy indicate the need for alternative drugs. The present study was designed to study the imatinib's targets (c-kit, VEGF, and PDGFR-α/β) expression in pituitary adenoma subtypes and elucidate the effects of imatinib on cultured human somatotropinoma cells and the rat somato-mammotroph GH3 cell-line and explore the plausible mechanism of action.

## Materials and methods

### Tissue microarray construction, staining, and image analysis

Following approval from Institute Ethics Committee of PGIMER, Chandigarh (Ref No INT/IEC/2016/2724), 157 cases of giant (maximum diameter ≥4 cm) pituitary adenomas [121 non-functioning pituitary adenomas (NFPA), 32 somatotropinomas, and four prolactinomas] were used for tissue microarray (TMA) construction. After-surgery, the cure rate of giant adenomas is less as compared to smaller adenomas and may require multi-modality treatment. None of the patients with prolactinomas had abnormalities of GH/IGF-1. Hematoxylin & eosin (H&E) staining was performed on paraffin section and the region of interest was identified. Two 3 millimeter cores were punched from each tumor and placed into a recipient block. Four non-adenomatous pituitary glands were taken at autopsy (within 4 h of death), from adult patients who died of non-endocrine disorders. Their sections were taken as control separately. None of the cases used in the study were known to harbor familial tumors or showed signs of syndromic disorder.

Both TMA and controls were stained simultaneously. The slides were deparaffinized in xylene, gradually rehydrated through a series of decreasing alcohols (100–70%) to distilled water and microwaved in 0.01 M sodium citrate buffer. Staining was performed with anti-PDGFR-α/β, anti-VEGF-A, anti-c-kit (Santa Cruz Biotech, California, USA), Ki-67, and anti-p53 (DAKO, USA) antibodies. Following washes in phosphate buffer saline (PBS), sections were incubated with the secondary antibody (anti-rabbit HRP conjugate, Santa Cruz). Staining was developed using the chromogen substrate diaminobenzidine (Liquid DAB, Dako K3468) followed by counterstaining with hematoxylin and rinsed in water. The brown signal obtained was visualized and scored under light microscopy. The stained slides were independently reviewed by three pathologists who were blinded for clinical and radiological details. Staining intensity and domain were scored as 0 (no staining), 1 (weak staining), 2 (moderate staining), and 3 (strong staining) according to a scoring system described previously by Tohti et al. (6).

### Double-immunofluorescence

Paraffin-embedded samples were sectioned at 7 μm thickness for histochemical evaluation. Double immunofluorescence was performed by deparaffinization of the sections followed by rehydration through decreasing ethanol dilutions. Heat-induced antigen retrieval was performed in 10 mM sodium citrate buffer (pH 6) with a microwave. Sections were left to cool down at room temperature and incubated for 1 h in blocking buffer [1X PBS, 0.1% Triton X-100, 5% Normal Goat Serum (Vector Laboratories, UK)]. Endogenous hPDGFR-α and hPDGFR-β were detected by staining the tissues overnight with a primary mouse monoclonal antibody against hPDGFR-α (Santa Cruz; sc-398206; 1:100) or PDGFR-β (Abcam; ab69506; 1:100). Staining against hGH was performed by incubating the tissues overnight with a primary rabbit polyclonal antibody against hGH. Overnight incubations with the primary antibodies were followed by 1 h incubation of the tissues with a secondary goat anti-mouse Alexa Fluor 488 antibody (Life Technologies; A11001; 1:200) in order to detect hPDGFR-α or hPDGFR-β. For the detection of hGH, tissues were incubated for 1 h with a secondary biotinylated goat anti-rabbit antibody (Vector Laboratories; BA-1000; 1:300) followed by DyLight 549 Streptavidin (Vector Laboratories; SA-5549; 1:300). Cell nuclei were stained with DAPI [4′,6-diamidino-2-phenylindole (VECTASHIELD Antifade Mounting Medium with DAPI; H-1200)]. Images were acquired using a confocal microscope (ZEISS LSM 880 with Airyscan) and figures were done using Adobe Photoshop CS6.

### Western blotting

Somatotropinoma (*n* = 27), NFPA (*n* = 27), prolactinomas (*n* = 4), and normal pituitary (*n* = 9) were homogenized in 1 ml of ice-cold PBS buffer containing 60 mM Tris–HCl, 1 mM EDTA (pH 6.8), and protease inhibitors. The lysate was then centrifuged at 10,000 × g for 10 min at 4°C. An aliquot of supernatant was taken to quantify proteins by the Pierce BCA protein assay kit (Thermofisher Scientific, MA, USA). Lysates (20 μg protein per sample) were resolved in 10% SDS-PAGE. The gel was then blotted onto a nitrocellulose membrane (Amersham, Aylesbury, UK) and probed with the corresponding primary antibody (VEGF 1:250, PDGFR-α 1:250, PDGFR-β 1:250, c-kit 1:250, Santa Cruz) followed by secondary antibody (bovine anti rabbit, Santa Cruz). Actin expression was evaluated to confirm equivalent total protein loading (rabbit anti-actin, 1:5,000, Sigma, Missouri, USA). Immunoreactive proteins were detected by electro chemoluminiscence (Amersham) using chemiluminescent imaging (FluorChem, Protein Simple, CA, USA). Intensity of the band was normalized to the corresponding actin intensity. The final data were subjected to gray-scale scanning and semi-quantitative analysis using Image J (National Institute of Mental Health, Maryland, USA). All samples were run in triplicates.

### Primary culture of human somatotropinoma and GH3 cell-line

Pituitary samples from 20 treatment-naïve acromegaly patients were used for this part of the study. The tumors were washed thoroughly with PBS (pH 7.4) until supernatant was clear and most red blood cells removed. The cells were dispersed using enzymatic (2.5% Trypsin, Gibco, USA) and mechanical dispersion procedure. Cells were washed with complete media and seeded in 24 and/or 96 well culture plate (Corning costar, USA). The primary culture was performed in Dulbecco Modified Eagle Media (DMEM, Gibco, USA) containing fetal calf serum (FCS, Gibco, USA), penicillin and streptomycin soon after transsphenoidal resection of the tumor. GH3 cells were obtained from American Type Culture Collection (ATCC, USA). These cells were grown in DMEM-F12 media (Gibco, USA) containing FCS. The cells were trypsinized and split once the confluence reached 80%. GH3 cells from the third passage were used for all experimental purpose. The cells were grown in complete media (DMEM + FCS + antibiotics) at 37°C and 5% CO_2_.

### Imatinib treatment

The stock solution of 50 mM of imatinib (Novartis Basel, Switzerland) were prepared by dissolving the compound in double distilled water and stored at −20°C until use. Cells were plated 12 h before imatinib was added at 0.25, 0.5, 1, 2.5, 5, and 10 μM concentrations. Effective dose 50 (ED_50_) was calculated by measuring the concentration of GH in culture supernatant. E-max method (four parametric logistic regression) was used to construct the dose-response curve and ED_50_ was calculated using the model built by substituting the dependent variable with half maximum value of GH. The effect of imatinib was compared to somatostatin analogs octreotide (0.1 μM; primarily SSTR2 agonist) and pasireotide (10 μM; SSTR1, 2, 3, and 5 agonist) with or without GHRH stimulation (0.5 μM) treated for 48 h.

### Growth hormone assay

Growth hormone was assessed from the primary culture supernatant using electro-chemiluminiscence immunoassay (COBAS, e601, Roche-Hitachi, USA) with lowest detection limit of 0.03 ng/ml and the inter and intra-assay coefficient of variation was < 2.5%. The human growth hormone differs structurally from the rat growth hormone. GH from GH3 cell line supernatant was measured using ELISA (Cloud-Clone Corp, SEA044Ra, Hubei, China).

### Immunocytochemistry

For immunocytochemistry, cells were cyto-spun onto poly-lysine double-coated slides at 1,000 rpm for 15 min and fixed with 4% paraformaldehyde. Excess formaldehyde was washed with PBS and the cells were exposed to rabbit anti-hGH antibody (1:400; DAKO) for 1 h and conjugated anti-rabbit anti-mouse secondary antibody (DAKO) for 30 min at room temperature. Thereafter cells were washed with PBS and incubated with DAB for 2 min followed by hematoxylin staining.

### Apoptosis and cell viability

Apoptosis was determined in untreated and imatinib treated GH3 cells by staining with annexinV-FITC antibody and propidium iodide (Molecular Probes, Life Technologies, USA) followed by flow cytometric analysis, measuring emission at 530 nm (FL1) and 575 nm (FL3).

The cell viability was measured by cellular uptake of 3-[4,5-dimethylthiazol-2-yl]-2,5-diphenyltetrazolium bromide (MTT). Cells were seeded at 4 × 10^3^ per well in 96 well plate containing 100 μl of culture medium with 10% FBS and cultured overnight. All analysis were performed in quadruplets. Prior to imatinib treatment, media was removed and fresh media was added. Subsequently, cells were exposed to imatinib. After 48 h of induction, 20 μl of MTT (0.05 g/ml, Himedia) was added to each well and incubated at 37°C for 4 h. After 48 h, the medium was removed, MTT solution was added and left for 4 h at 37°C. The medium was aspirated and DMSO was added to each well. The color development was measured at 570 and 630 nm (Infinite M200 pro, Tecan, Austria). The absorbance was proportional to the number of viable cells.

% Cell Viability = Optical density of imatinib treated cells/Optical density of untreated cells × 100.

### Electron microscopy

Both treated and control GH3 cells were harvested in 2.5% glutaraldehyde and were washed with Sorenson buffer, millonin buffer and 1% osmium tetroxide. Thereafter, cells were dehydrated in graded series of ethanol, processed through propylene oxide, and embedded in an Epon-araldite mixture (Epon 812, Sigma, USA). Ultrathin sections were stained with uranyl acetate lead citrate and viewed under the electron microscope (JEM 1400 Flash, JEOL, USA).

### Receptor tyrosine kinase array

To investigate the mechanism of GH inhibition by imatinib we used human phospho-receptor tyrosine kinase (RTK) array to screen 49 different phospho-RTKs for differentially activated kinase in response to imatinib treatment in somatotropinoma cells. Cell lysate from both treated and control somatotropinoma cultured cells were extracted and subjected to receptor tyrosine kinase array (R&D Systems, MN, USA) according to manufacturer's protocol. The kit is specifically designed to screen relative level of phosphorylation of 49 different RTK. Phosphorylation status was determined by chemiluminescence and analyzed using Image J software (National Institute of Mental Health, Maryland, USA).

### Statistical analysis

The statistical analysis was performed using SPSS version 16. For two group comparisons Student's *t*-test or Mann–Whitney *U*-test and for multiple comparison one-way ANOVA (followed by Bonferroni *post-hoc* test) or Kruskal–Wallis test (followed by Dunn's test) was used depending of the normal distribution of the data. Spearman's test was used for correlations. *P*-value of < 0.05 was considered as significant.

## Results

### Low dose of imatinib attenuates gh secretion without affecting cell number and viability

**Figure 1:**
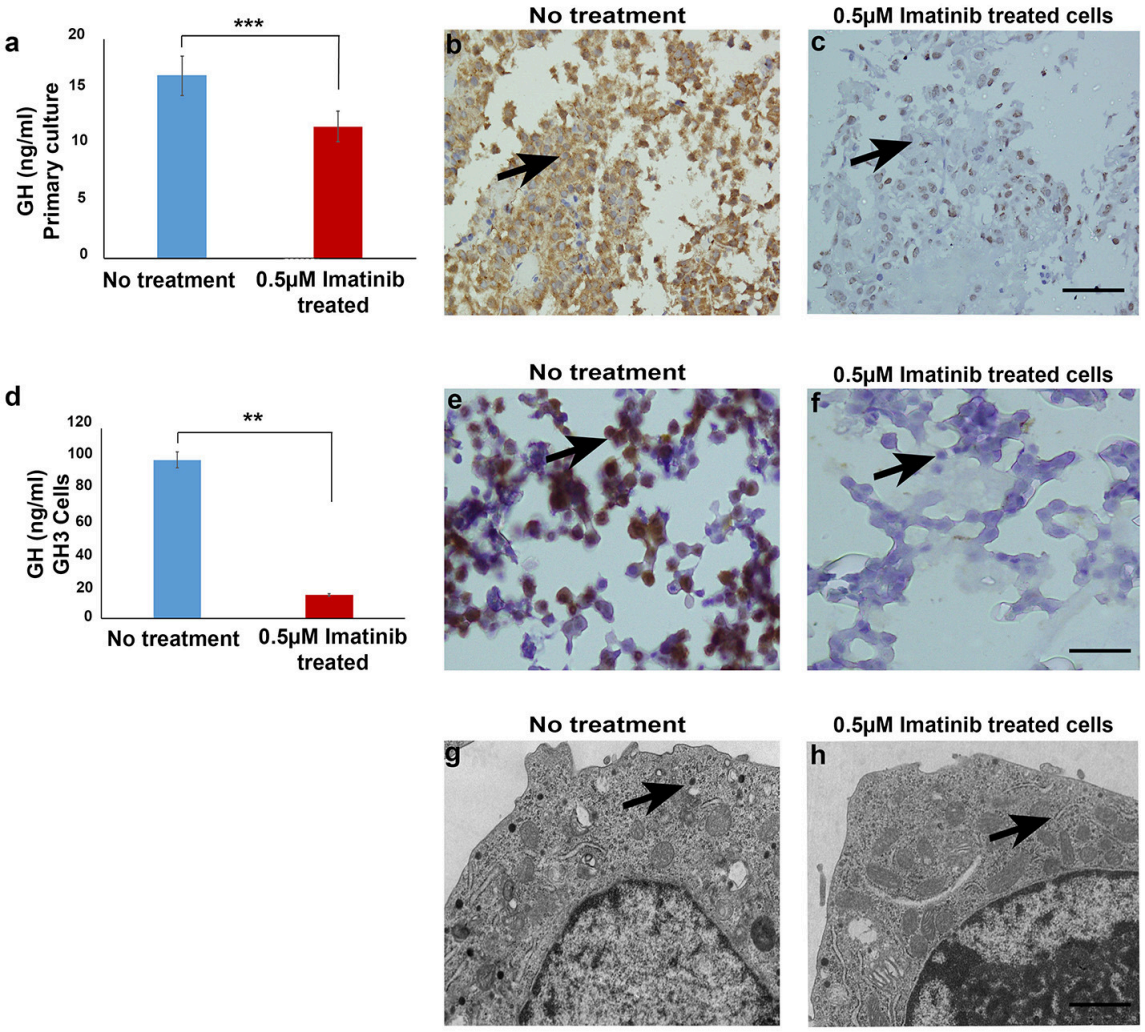
**(a)** Bar diagram showing reduction in growth hormone levels in culture media after treatment with 0.5 μM imatinib in primary culture of somatotropinoma cells (*P* < 0.0001). **(b,c)** Representative image of immunohistochemistry with anti-GH antibody on treated and untreated primary somatotropinoma culture. **(d)** Bar diagram showing reduction in GH levels in culture media of GH3 cell line after treatment with 0.5 μM imatinib (*P* < 0.01). **(e,f)** IHC of GH3 cells showing intense GH positivity in untreated and no GH staining in treated cells (20X). **(g,h)** Ultra-structural analysis of GH granules using electron microscopy between treated and untreated GH3 cells (6000X). **P < 0.01; ***P < 0.0001.

To assess the effect of imatinib treatment of GH secretion, primary cultured cells from 20 somatotropinomas were tested (patient characteristics are shown in Table 1). Cells were treated with increasing concentrations of imatinib (0.5–10 μM) and levels of GH measured using electrochemoluminiscence, cell viability was measured using MTT assay (Supplementary Figure 1). The maximum reduction in GH was observed at concentration of 0.5 μM of imatinib, with statistically significant reduction at higher doses (1–10 μM). The mean GH measured from the 20 different somatotropinoma primary cultures treated with 0.5 μM of imatinib showed 20% reduction in GH compared to controls (*p* < 0.001; Figure 1a). To further assess the inhibitory effect on GH secretion, pituitary rat tumor cells line GH3 (GH and PRL secreting) were treated with 0.5 μM imatinib. After treatment, 88% reduction of GH secretion was observed (*p* < 0.001; Figure 1d). These results were further confirmed by immunostaining against GH in both primary tumor and GH3 cultured cells. 0.5 μM imatinib treated cells exhibited negligible GH staining in both primary culture and GH3 cells (Figures 1b,c,e,f, respectively). We then assessed if the imatinib treatment had and effect on cell proliferation, viability, and apoptosis of GH3 cells. Cell viability was studied post imatinib treatment using cellular uptake of MTT. No significant differences in cell viability or proliferation (*p* < 0.88) were observed between treated and untreated cells (Supplementary Figure 2). Moreover, the viability analysis at 0, 24, and 48 h also showed no change in somatotropinoma viability. To assess if imatinib leads to inhibition of GH secretion due to reduced *GH* and *Pit-1* transcription we used real time PCR from primary somatotropinoma cultures. Treatment with imatinib did not affect *GH* or *Pit-1* transcripts (Supplementary Figure 3). This prompted us to study if the secretion of GH vesicles was affected in GH3 tumor cells after imatinib treatment using transmission electron microscopy (TEM). Interestingly, untreated cells showed secretory granules of 122–150 nm localized to Golgi region and the periphery, whilst cells treated with 0.5 μM imatinib showed marked reduction in granularity of secretory vesicles (Figures 1g,h). Due to paucity of the cells, similar results could not be reproduced in imatinib-treated cells from primary culture. Together this data indicates that imatinib reduces GH secretion by reducing the secretory granules.

**Table 1:** Patient characteristics for primary culture of somatotropinoma.

**Parameters**	**Value**
Gender (M:F)	10:10
Mean age (years)	33.1 ± 13
Mean tumor volume (mm^3^)	10672.82
Mean pre op GH (ng/ml)	25.09 ± 22.68
Mean pre op IGF-1 (ng/ml)	872.09 ± 352.22
**IMMUNOHISTOCHEMISTRY**	
GH+	13
GH+ Prl+	7

### Expression of imatinib targets (c-kit, PDGFR-α, PDGFR-β, and VEGF) in pituitary adenomas using immunostaining

**Figure 2:**
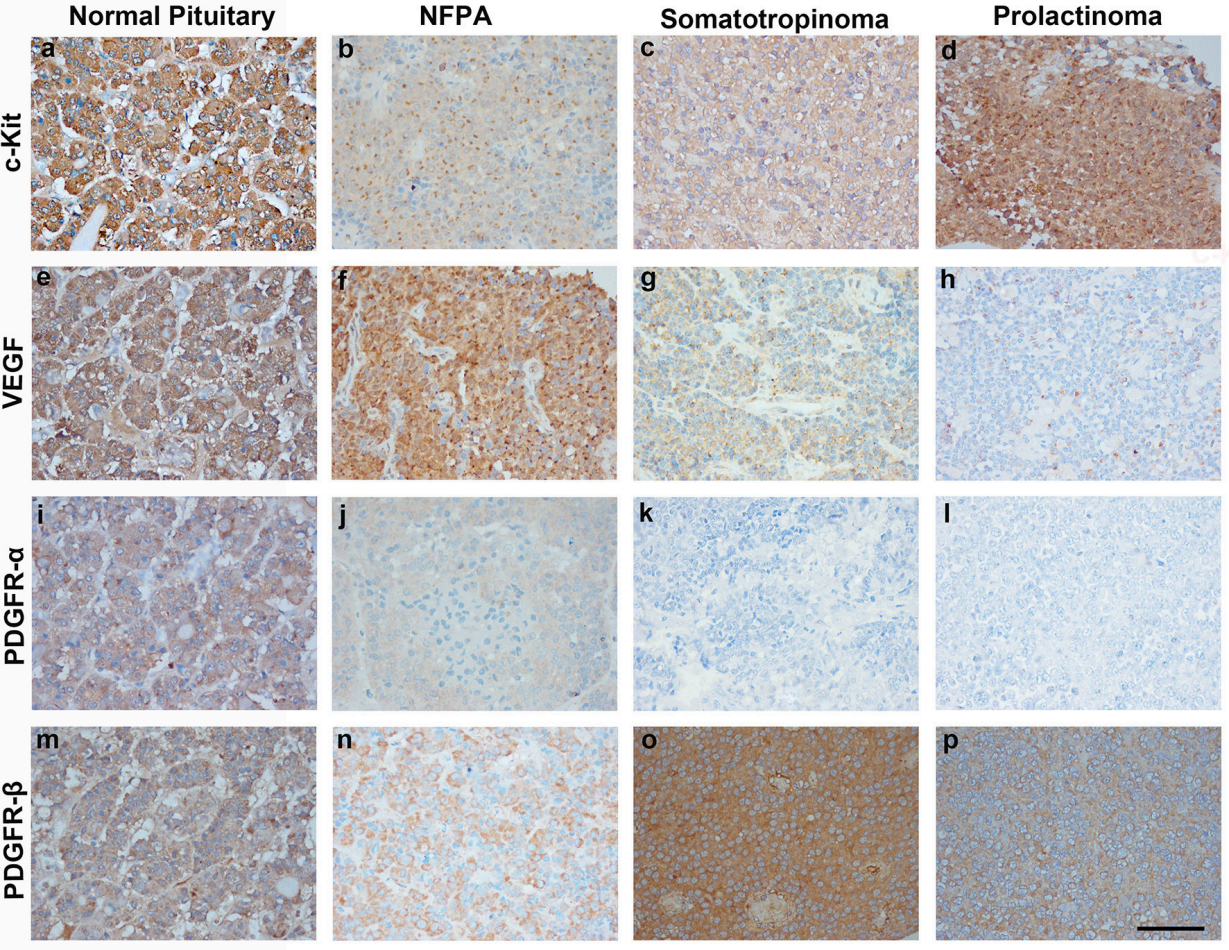
Immunohistochemistry (IHC) of pituitary adenoma shows differential expression of tyrosine kinases (c-kit, VEGF, and PDGFR-α/β). Cytoplasmic immunopositivity for c-Kit was high in somatotropinoma followed by NFPA, prolactinoma, and normal pituitary **(a–d)**. Strong cytoplasmic expression for VEGF was observed in NFPA as compared to somatotropinomas, prolactinomas and normal pituitaries **(e–h)**. PDGFR-α expression was weakly positive in NFPA as compared to normal pituitary while it was negative for somatotropinoma and prolactinomas. **(i–l)**, PDGFR-β expression was strongly positive in somatotropinomas as compared to normal pituitary while it was weakly positive in NFPA and prolactinomas **(m–p)**.

**Figure 3:**
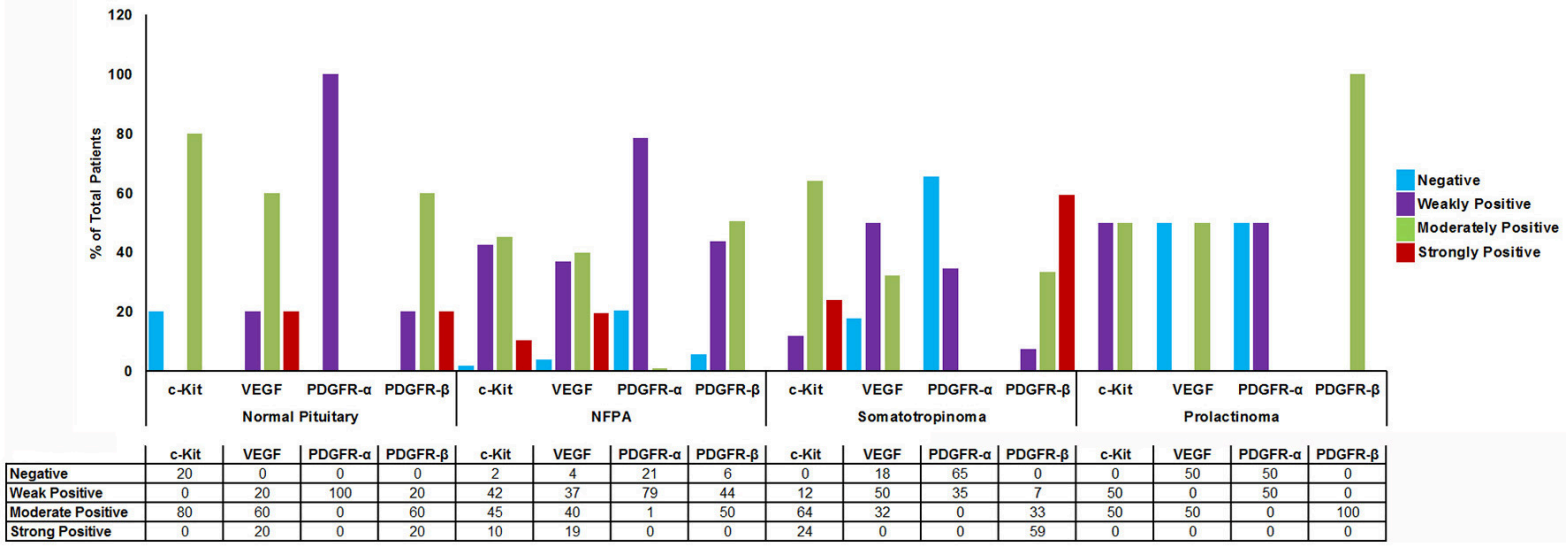
Graph showing percentage of cytoplasmic TK (c-Kit, VEGF, PDGFR-α/β) immunostaining in normal pituitary (*n* = 4), NFPAs (*n* = 121), somatotropinomas (*n* = 32), and prolactinomas (*n* = 4). c-Kit showed moderate to strong positivity in somatotropinomas. VEGF showed higher percentage of positivity in NFPA. PDGFR-β was strongly positive in maximum number of somatotropinomas (59%), whereas PDGFR-α was negative (65%).

Imatinib has been shown to inhibit tyrosine kinases activity in several tumors (7). In particular it has been shown to inhibit the tyrosine kinase c-Kit, PDGFR-α, PDGFR-β, and VEGF. The differential expression of imatinib targets were analyzed in a cohort of 157 patients with giant pituitary adenomas (>4 cm). The patient characteristics are shown in Table 2. c-Kit was expressed in a large proportion of pituitary adenomas (100% in somatotropinomas and prolactinomas and 97% in NFPAs; Figures 2, 3). In patients with somatotropinomas, moderate to strong positivity was much more frequent as compared to NFPAs (88 vs. 55%, *P* = 0.009). No difference was found when compared to normal pituitary for both adenomas. PDGFR-α positivity was observed in all controls, 79% NFPA, 50% prolactinomas and only 35% somatotropinomas (Figures 2, 3). The PDGFR-α cytoplasmic staining showed significant increase in NFPA, compared to somatotropinoma (*P* < 0.001). As compared to the controls, the PDGFR-α was poorly expressed in somatotropinoma (100 vs. 35%, *P* = 0.03). The PDGFR-β was expressed in all adenoma subtypes as well as controls, with somatotropinomas showing higher expression compared to NFPA or control (59 vs. 20%, *P* < 0.001; Figures 2, 3). Strong PDGFR-β positivity was observed in somatotropinomas as compared to control. VEGF expression was observed in both normal (100%) and adenomatous pituitaries. Overall, VEGF positivity was identified in 96% NFPA, 82% somatotropinomas and 50% prolactinomas (Figures 2, 3). However, strong VEGF positivity was observed in NFPA more often than somatotropinomas (19 vs. 0%, *P* < 0.003) but no difference was observed when compared to the controls.

**Table 2:** Patient characteristics for tissue microarray staining.

	**NFPA (*n* = 121)**	**Somatotropinoma (*n* = 32)**	**Prolactinoma (*n* = 4)**
Gender (Males: Females)	74 (61.1%): 47 (38.8%)	20 (62.5%): 12 (37.5)	3 (75%): 1 (25%)
Mean age (years)	43.1 (±12.6)	33.2 (±11.1)	33.0 (±17.1)
Complete resection	47	9	4
Residual lesion	74	23	0
Underwent second surgery	21	5	0
Post-op radiotherapy/gamma knife	18	12	0
**IMMUNOHISTOCHEMISTRY**			
Non-functioning adenoma (all six negative)^*^	95.9%	3.1%^**^	0
**LH/FSH POSITIVE**			
GH +ve	1.7%	65.6%	0
GH and PRL+ve	2.4%	31.2%	100%

* *They were not stained for α-subunit of gonadotropin*.

** *Negative for GH, LH, FSH, ACTH, and PRL staining*.

Comparison between various tyrosine kinase pathway members in different pituitary adenomas are enlisted in Figure 3. Though all patients had giant pituitary adenoma, they were not overtly proliferative (Ki-67 < 3% in all). There was no correlation between TK pathway members expression (VEGF, c-kit, PDGFR-α/β), immuno-positivity to pituitary adenoma subtypes and Ki-67, p53, age, gender, recurrent, or residual lesion (data not shown).

### Co-localization of GH and PDGFR-α/β in somatotropinomas and normal pituitary

**Figure 4:**
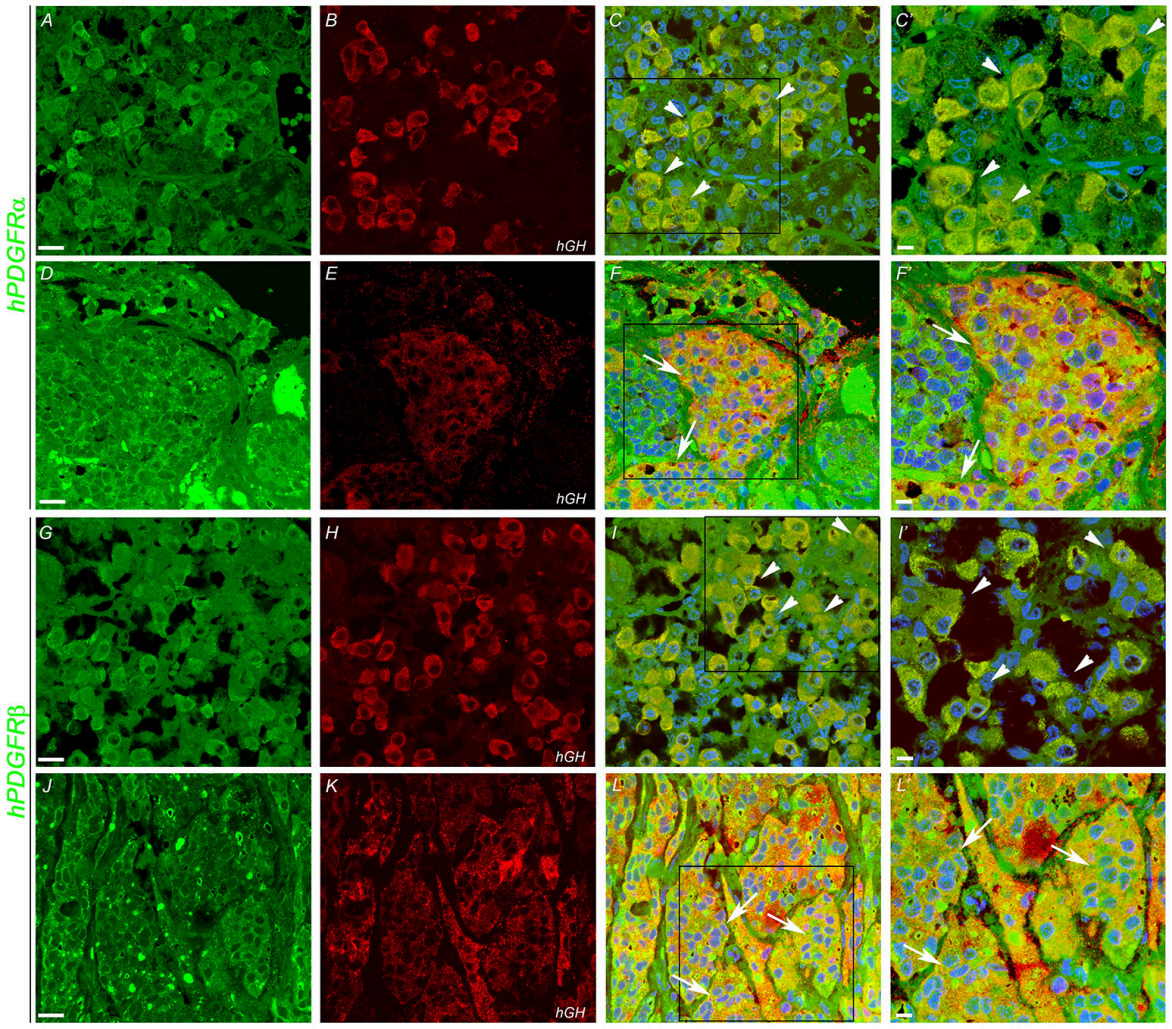
Double immunofluoroscence showing co-expression (arrows) of PDGFR-α and PDGFR-β (green) and of hGH (red) in normal pituitary gland **(A,B,G,H)** and in somatotropinoma **(D,E,J,K)**. Overlays of the green and red channels are shown in the third column. **(C**′**,F**′**,I**′**,L**′**)** are the magnified views of the boxes marked in **(C,F,I,L)** respectively. Scale bar = 100 μm **(A–L)**, 50 μm **(C**′**,F**′**,I**′**,L**′**)**.

In order to determine which cell type expresses imatinib targets, double immunofluorescence staining was performed with anti-GH, anti-PDGFR-α, and PDGFR-β on somatotropinoma and normal pituitary tissues. Colocalization between GH and both PDGFR-α/β was observed in both normal pituitary and somatotropinoma cells. There was loss of PDGFR-α and gain of PDGFR-β in somatotropinomas (Figure 4). This data shows that GH cells express the imatinib target PDGFR-α/β.

### Quantitative analysis of c-kit, PDGFR-α, PDGFR-β, and VEGF

**Figure 5:**
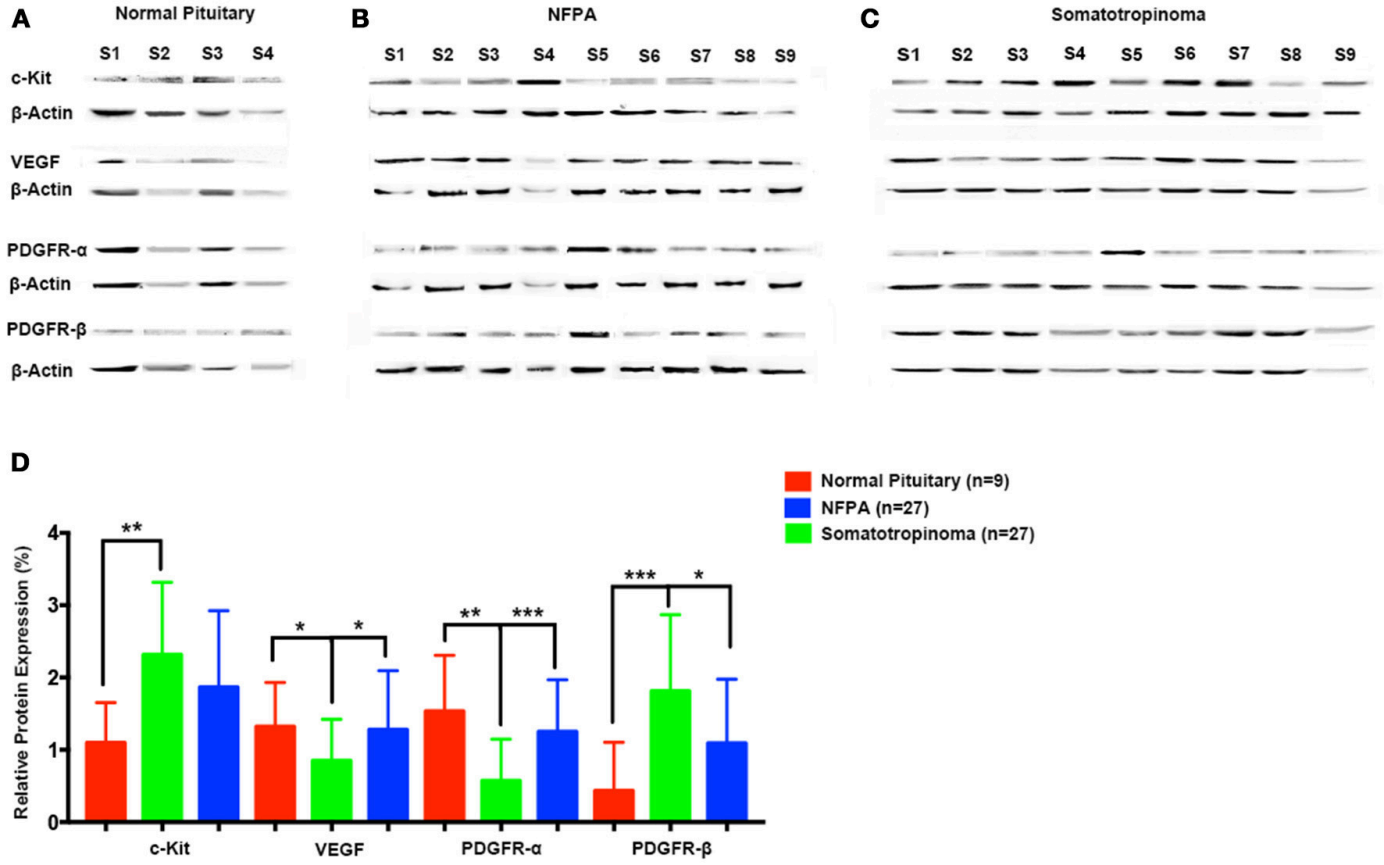
Quantification of tyrosine kinases (c-Kit, VEGF, PDGFR-α, and PDGFR-β) using western blot in **(A)** normal pituitary (*n* = 9), **(B)** NFPA (*n* = 27), and **(C)** somatotropinomas (*n* = 27). Data were normalized to β-Actin and compared to expression in normal pituitary. **(D)** Similar to IHC findings, PDGFR-β and c-Kit was overexpressed in somatotropinoma (*P* < 0.01) compared to normal pituitary whereas PDGFR-α was under expressed in somatotropinoma (*P* < 0.05). *P < 0.05; **P < 0.01 and ***P < 0.001.

To quantify the differential expression between tumor subtypes and imatinib targets, we evaluated protein expression of c-Kit VEGF, PDGFR-α, and PDGFR-β in somatotropinomas (*n* = 27), NFPA (*n* = 27), prolactinomas (*n* = 4), and normal pituitaries (*n* = 9) using western blotting (Figure 5). As compared to normal pituitaries, c-Kit protein expression was found to be significantly higher in somatotropinomas (224.3%, *P* < 0.05) and NFPA (193.5%, *P* < 0.05). Notably, somatotropinoma cells exhibited higher expression of PDGFR-β protein when compared to normal pituitary tissue (195.8 vs. 100%, *P* < 0.05). Compared to normal pituitary, the relative expression of PDGFR-α was found to be lower in somatotropinomas (37.1%, *P* < 0.05) and NFPA (81.4%, *P* = 0.08). Hence, these data indicate a relative over expression of PDGFR-β and under expression of PDGFR-α in somatotropinomas. VEGF was differentially expressed between somatotropinomas and NFPA (68.2 vs. 104.7%, *P* < 0.03), compared to normal pituitary (100%). However, no correlation was observed between TKs protein expression in both adenomas. Our quantification studies indicate that PDFGR-β is unregulated in somatotropinaomas compared to both NFPAs and normal pituitary.

### Efficacy of imatinib compared to gold standard drugs—octreotide and pasireotide

The somatotropinoma cells were exposed to different concentration of GHRH (0–10 μM), octreotide (0–10^−9^ M) and pasireotide (0–20 μM). After standardization, the effect of imatinib on somatotropinomas was compared with octreotide (0.1 μM), pasireotide (10 μM), and GHRH (0.5 μM). It was observed that, compared to vehicle-treated controls, GHRH stimulation enhances GH synthesis (125%, *P* < 0.01) whereas pasireotide and octreotide cause significant decrease in GH (39 and 45.8%, *P* < 0.0001 and *P* < 0.003, respectively; Supplementary Figure 2). Although imatinib causes GH reduction in somatotropinoma cells (30%, *P* < 0.01), it was found to be less efficient than pasireotide (54%, *P* < 0.01) and octreotide (64%, *P* < 0.01; Supplementary Figure 4).

### Mechanism of imatinib inhibition of GH secretion

**Figure 6:**
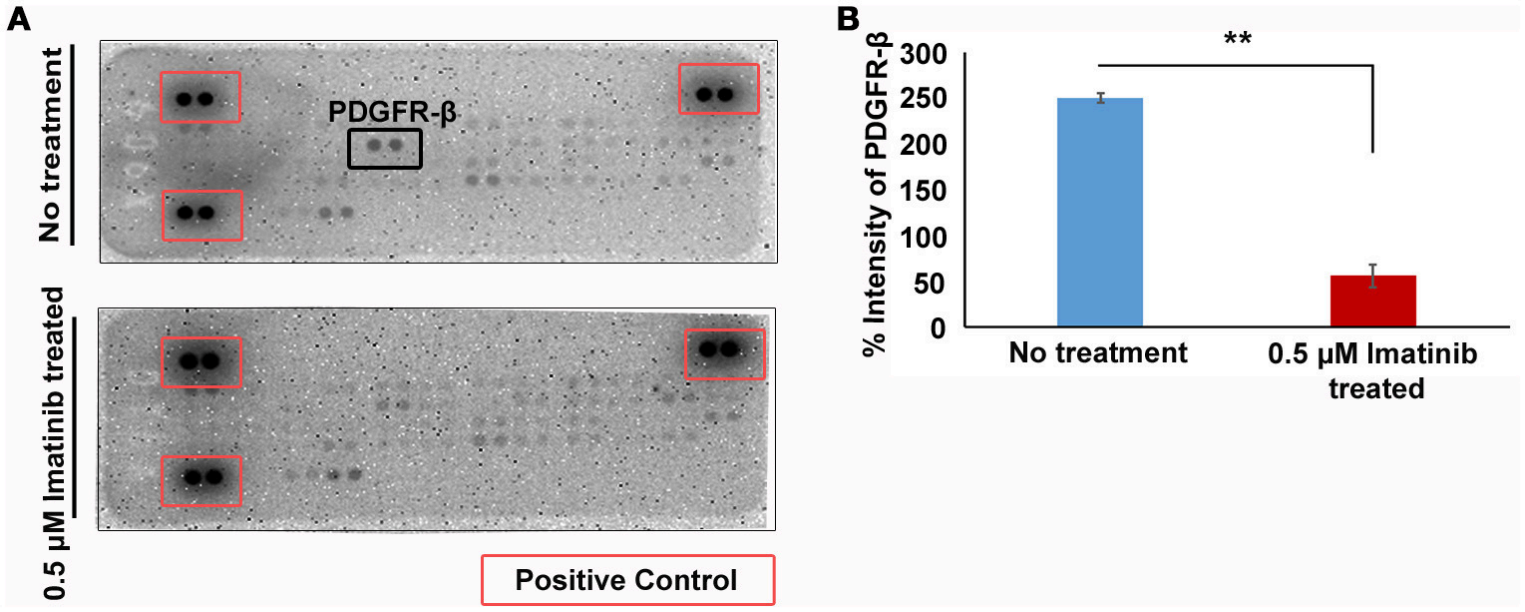
Phospho-tyrosine profiling of somatotropinomas treated and untreated with 0.5 μM Imatinib. **P < 0.01. **(A)** Phospho-tyrosine profiling of somatotropinomas treated and untreated with 0.5 μM Imatinib. Note the absence of PDGFR-β after imatinib treatment **(B)** Bar diagram showing reduction in PDGFR-β levels after treatment with 0.5 μM imatinib in primary culture of somatotropinoma cells (*P* < 0.01).

Our data indicates that imatinib has inhibitory effect on GH secretion in somatotropinomas. In order to understand the mechanism of action of imatinib in GH reduction in somatotropinoma cells we employed a tyrosine kinase (RTK) array. Cell lysates from somatotropinomas were subjected to tyrosine kinase array. Importantly we identified that PDGFR-beta was significantly decreased in the imatinib treated cell compared to the untreated cells. Importantly, this suggests that imatinib-mediated inhibition of GH release is mediated through PDGFR-β activation and the array detect phosphorylation status of the receptors (Figure 6).

## Discussion

In this study, we have demonstrated the differential expression of imatinib targets (c-kit, PDGFR-α/β, and VEGF) in different pituitary adenomas subtypes and we have shown that imatinib decreases GH secretion in cultured somatotropinoma cells and GH3 cell line acting through PDGFR-β pathway.

In the present study, we found differential expression of TK pathway members (c-kit, VEGF and PDGFR-α/β) on a large cohort of pituitary adenoma subtypes compared with normal pituitary samples. We have shown that in somatotropinomas and prolactinomas there is increased positivity of c-kit and PDGFR-β, whereas in NFPAs exhibit higher VEGF and PDGFR-α positivity. Thus, it was speculated that prolactinomas and somatotropinomas lose PDGFR-α and acquire PDGFR-β. This result could have potentials for more precise mechanistic insights and therapeutic approaches.

La Rosa et al. have evaluated c-kit expression in normal human pituitary and 62 well characterized pituitary adenomas. In their study, c-kit expression was predominantly found in corticotropinomas followed by NFPA. However, it was absent in prolactinomas and somatotropinomas (8). Unlike their study, we found predominant expression of c-kit in NFPA and somatotropinomas, though we have not examined any patient with corticotropinoma and the intensity of positivity varied from tumor subtypes. Similar to our study, Casar et al. found cytoplasmic c-kit positivity in 52.4% and membranous positivity in 8.3% (9). Usually it is believed that the mutated protein translocates into the nucleus and gives nuclear positivity. However, neither our study nor Casar et al. had identified any mutations in c-kit (10). We did whole exome sequencing in both in blood and tumor tissue in 11 patients with acromegaly and didn't find any mutation of c-kit (data not shown). Platelet-derived growth factor (PDGF) is a potent mitogen known to stimulate tumor growth in a number of human tumors through autocrine and/or paracrine loops (11). PDGF receptor and its ligand are believed to be expressed predominantly in folliculo stellate cells of the pituitary gland and regulate VEGF expression and activity (12). In a study by Sullivan et al. PDGF has been reported to stimulate GH secretion in somatotrophs and inhibit prolactin release from lacto-somatotroph GH4C1 and GH3 cell-lines (13). However, little is known about the expression of PDGFR in pituitary adenomas. In our study, the expression of PDGFR isoforms varied greatly between the pituitary tumor subtypes. Our results showed that PDGFR-α was over-expressed in NFPA compared to somatotropinomas, whereas PDGFR-β was increased in somatotropinomas as compared to NFPA. Double immunofluorescence confirmed reduced expression of PDGFR-α and increased expression of PDGFR-β. However, quantification by western blot showed definite increase in PDGFR-β levels.

The VEGF family consists of five glycoproteins (VEGF A-D) as well as placental growth factor. Of these, VEGF-A is the best characterized and commonly referred to as VEGF in humans (14). There have been contradicting reports about VEGF expression in pituitary adenomas. A study from McCabe et al. has shown that VEGF mRNA expression is higher in pituitary adenomas compared to the normal pituitary gland, probably due to PTTG action (15). Lloyd et al. have reported stronger VEGF expression in normal pituitary as compared to pituitary adenomas (16). Moreover, another report showed no significant difference in VEGF immunostaining between normal and adenomatous pituitary gland (17). However, decreased VEGF expression in pituitary adenomas has also been reported by Raica et al. (18). Anti-angiogenic therapy can sensitize tumor stem cells to radio- and chemo-therapy. Inhibition of the VEGF pathway can be achieved via neutralizing antibodies against VEGF (19). The VEGF receptor (VEGFR) expression is poorly studied in normal and adenomatous pituitary. VEGFR-2 expression analyzed on rodent showed elevated expression of VEGFR-2 after estrogen treatment (20). In our cohort, we observed significantly high expression of VEGF in NFPA as compared to somatotropinomas. Consistent with the results from previous study by McCabe et al. (15). VEGF was found to be differentially expressed in adenomatous pituitary in our cohort also (18). Like in our study, the work done by Cristina et al., showed that all our patients with prolactinoma (though small in number) were VEGF positive (21). Further, they have also shown that expression of above mentioned TK pathway members were not influenced by gender, age and Ki-67 index (21).

*In vitro* samples of primary cultures of somatotropinoma and rat pituitary adenoma cell line (GH3) showed that imatinib, a drug that causes growth failure in CML probably targeting GH or IGF-1 axis, inhibited GH secretion in a dose-independent manner and without affecting cell viability. GH lowering response was much robust in GH3 cell line, compared to primary culture. This could be because of pure cell population in case of commercial cell line. Our results also shed light on the mechanism of action of imatinib, which acts by inhibiting GH signaling via PDGFR-β/PKC pathway. However, it does not causes apoptosis. Further, real time PCR for *Pit-1* and *GH1* genes showed that imatinib does not affect GH synthesis but inhibits GH secretion, which is also corroborated by electron microscopic findings of loss of GH secretory granules in imatinib-treated GH3 cells. Similar to our cell viability results, a previous study by Venalis et al. have also shown that imatinib does not affect proliferation, viability, migration and metabolic activity of endothelial cells (22). At low concentration, it has relatively similar inhibition of BCR-ABL and c-kit in various malignancies. In patients with CML treated with 400–600 mg imatinib per day, the plasma concentration of 0.17–5.68 μM shows cytogenetic and hematologic response. The ED50 of imatinib calculated from our experiment was 0.015 μM, which is lower than the clinically relevant plasma concentrations (Supplementary Figure 5). Therefore, the plasma concentrations that can be achieved are likely to inhibit GH synthesis enough to be beneficial in patients with somatotropinoma. The maximum achievable plasma levels of imatinib in patients are not higher than 6.7 μM at maximum administered dose of 600 mg per day. Higher level of plasma concentration of imatinib is difficult to achieve because of adverse side effects of the drug. However, extrapolation of the data obtained from *in vitro* model to real life situation in patients with acromegaly needs long term trials. Although octreotide and pasireotide are more efficacious, imatinib could be used as an inexpensive alternative therapy or as an adjunct.

Patients with childhood CML who are on imatinib (targets-c-kit, PDGFR-α/β, and VEGF) and are in remission have growth retardation by affecting GH/IGF-1 axis, probably acting on somatotrophs (5). Previously we have shown a close association in common pathogenic mechanism in acromegaly and hematological disorders (23). Our observation of use of anti-VEGF therapy in childhood somatotropinomas demonstrated that it was very effective (24). Similarly, Ortiz et al. have successfully used the anti-VEGF antibody in a patient with silent corticotroph carcinoma with successful outcome (25). So, it could be a therapeutic option in selected subgroup of aggressive pituitary adenoma with or without other TKIs. Increased expression of VEGF in tumors that undergo apoplexy is well-known (26). We presume that this can be tried in future as a therapeutic option in patients with pituitary apoplexy who are poor surgical candidates, akin to vitreous hemorrhage in patients with diabetic retinopathy. A previous study by Fukuoka et al. have shown successful use of gefitinib, an epidermal growth factor receptor (EGFR) tyrosine kinase inhibitor in human and canine cultured corticotropinoma (27). They also reported that in mice, gefitinib treatment decreased both tumor size and cortisol level. However, mere expression of receptor in a tumor seen on IHC does not necessarily mean response to therapies targeting these receptors. Thus, NFPA express somatostatin receptor type 2 (SSTR2) and somatostatin receptor type 5 (SSTR5), they poorly responded to somatostatin analogs (28).

The cross-talk between different hormones, cytokines, receptors and growth factors play an important role in regulating cellular response. The GH receptor and the downstream pathways share a complex relationship with other receptor and signaling pathway members. A variety of signaling pathways, including the Src, Grb2, MEK/MAP kinase, the phosphatidylinositol 3 (PI-3)-kinase, and the protein kinase C (PKC) pathways has been shown to be activated by both GH and PDGF. GH has been shown to interact with the IGF-1 receptor and downstream members of IGF-1 signaling pathways (29). A study by Rui et al. has reported that PDGF down regulate GH signaling via PKC dependent pathway. Further, they have shown that PDGF significantly reduces the tyrosyl phosphorylation of GHR (by 90%) and the amount of both total cellular GHR (by 80%) and GH binding (by 70%) (30). In somatotropinomas, both GH/GHR and PDGF/PDGFR can synergize the signal transduction that they elicit, at least in part by virtue of GH's ability to potentiate and sustain GH signaling. We hypothesize that imatinib might inhibit PDGFR-β preventing the binding of PDGF to PDGFR-β, thereby increasing the extracellular concentration of PDGF which further inhibits GH signaling pathway. In future we need to prove by adding PDGFR beta ligand to primary culture and see that GH is stimulated. On the other way PDGFR-β can be absorbed with an antibody and the cells can be thwart with this mixture to show that the antibody takes away the release effect of PDGFR-β. The somatostatin receptor ligands also inhibits GH through PKC and downstream pathway. In pediatric CML cases, growth retardation due to imatinib could be through this pathway.

The strength of our study is large sample size for validation through IHC, *in vitro* culture using both somatotropinoma cells and GH3 cell line. Four tire validation of protein expression doing IHC, western blot, RTK array, co-localization using double immunofluorescence and negative and positive controls using brain, tonsilar and placental tissue to exclude non-specific antibody binding (Supplementary Figure 6).

The limitations of our study are use of TMA which can sometimes not represent the expression of the whole sample due to heterogeneity. The second limitation is we had not looked for the impact of imatinib in densely and sparsely granulated somatototroph adenomas of variable sizes. We would have been wiser looking at the combined effect of imatinib and octreotide/pasireotide to look for augmented effect if any. Last but not the least data on PDGF stimulation and inhibition could have strengthened the study.

In conclusion, Imatinib targets (c-kit, VEGF, PDGFR-α/β) are differentially expressed between various giant pituitary adenoma subtypes and can serve as potential biomarkers. PDGFR-β was found to be over-expressed in giant somatotropinomas and possibly be used as selective target. Imatinib reduces GH secretion both in GH3 cell line and cultured somatotropinoma cell *in vitro* without affecting cell number. Therefore, it could be used as an adjunct for treating GH secreting pituitary adenoma.

Way forward: A clinical trial in a large cohort showing the response of somatotropinoma of variable sizes both in sparsely and coarsely granulated tumors to imatinib is required. Similarly, we have to weigh the risk and benefits of this drug because of potential side effects before it can be recommended for clinical use. Multi-targeted TKIs might be a suitable alternative treatment for these tumors in desperate cases.

## Ethics statement

The Study was carried out in accordance with the recommendations of Institute Ethics Committee of Postgraduate Institute of Medical Education and Research (PGIMER), Chandigarh, India. All subjects gave written informed consent in accordance with the Declaration of Helsinki. The protocol was approved by Institute Ethics Committee of PGIMER, Chandigarh, India (Ref No INT/IEC/2016/2724).

## Author contributions

All authors listed have made a substantial, direct and intellectual contribution to the work, and approved it for publication.

### Conflict of interest statement

The authors declare that the research was conducted in the absence of any commercial or financial relationships that could be construed as a potential conflict of interest.

## Tribute

We are deeply saddened to inform the sudden, untimely and tragic demise of Prof. K.K. Mukherjee, Department of Neurosurgery, PGIMER, Chandigarh at the end of the study before publication could see the light. He was our dear friend, senior colleague and mentor. May his soul rest in peace and we wish that his legacy would continue.
