# Supplementary material for: Imatinib Inhibits GH Secretion From Somatotropinomas
Source: Front Endocrinol (Lausanne). 2018 Aug 27;9:453. doi: 10.3389/fendo.2018.00453 (PMC6120347; doi:10.3389/fendo.2018.00453)
Supplement: Supplementary file 1 [file Presentation_1.pdf]

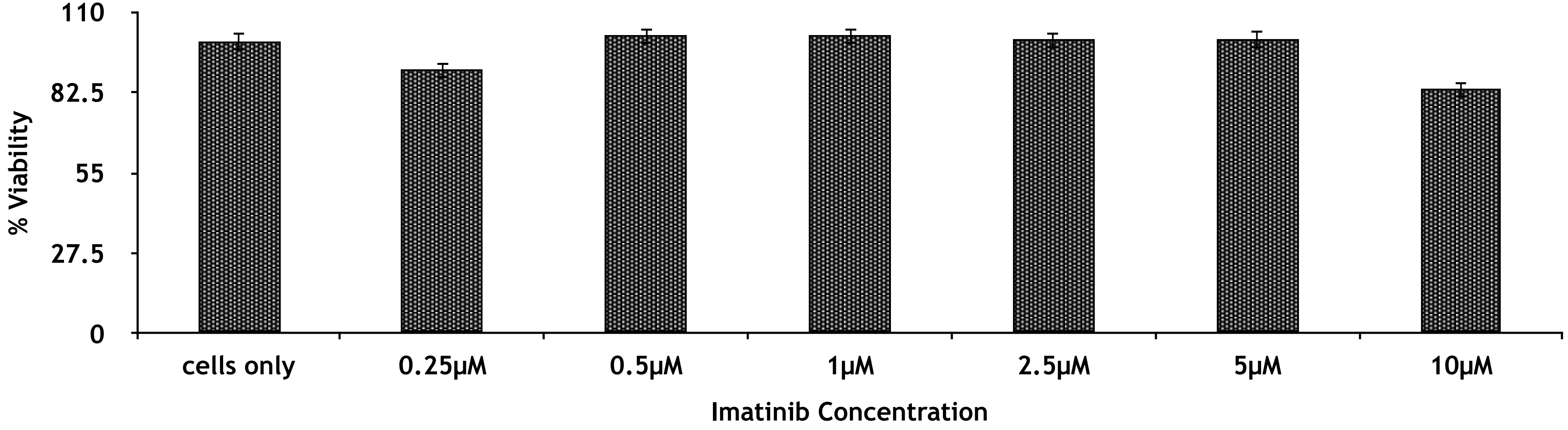

Figure 1: Graph showing cell viability with different concentration of Imatinib

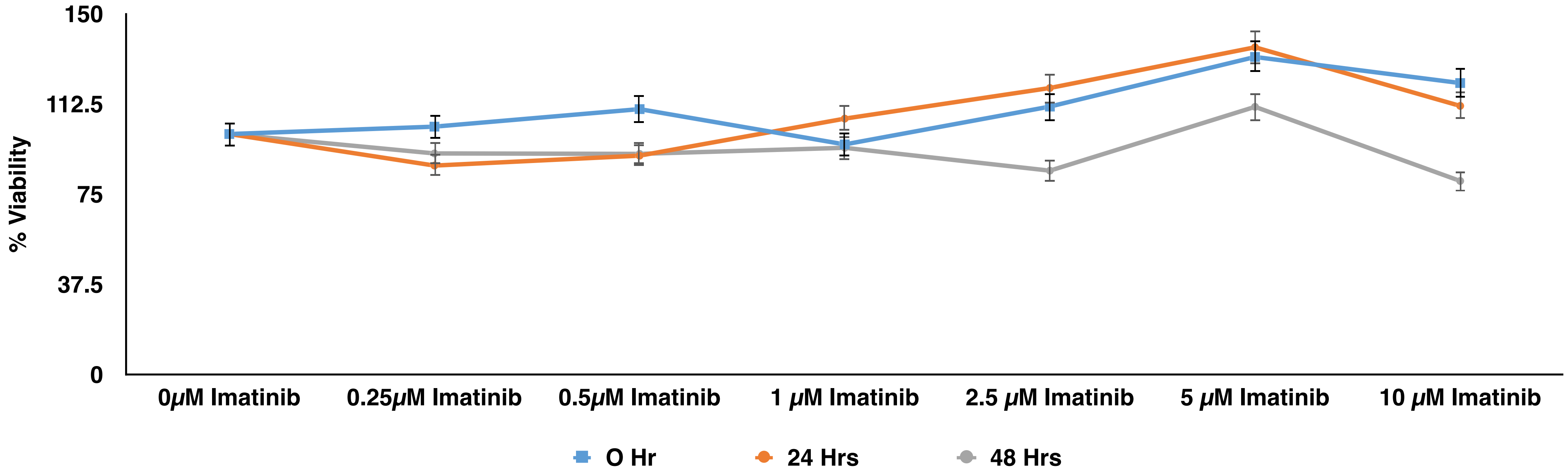

Figure 2: Graph showing cell viability assay at 0, 24h and 48h.

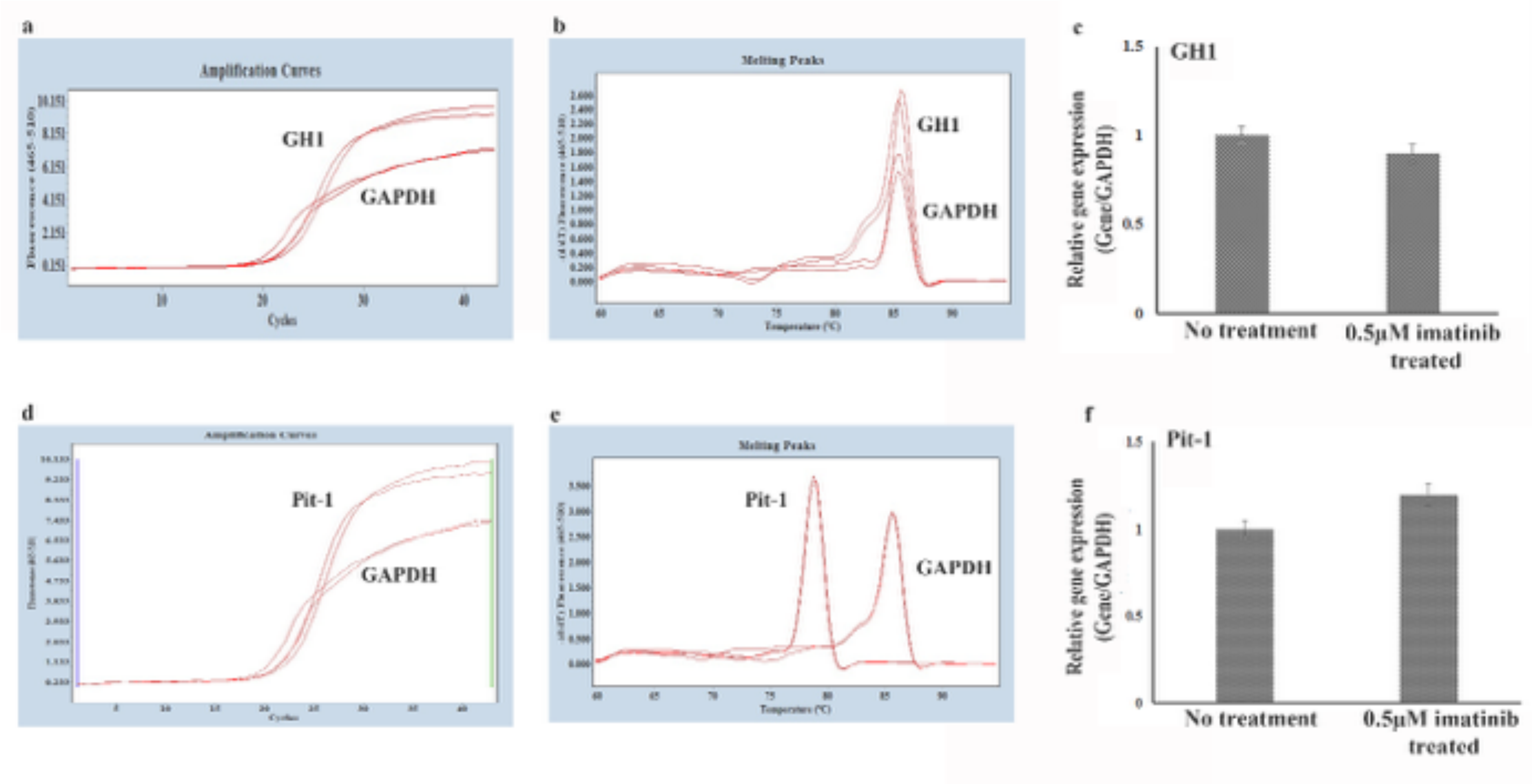

Figure 3: Real-time PCR analysis of effect of imatinib on *Pit-1* and *GH1* gene.

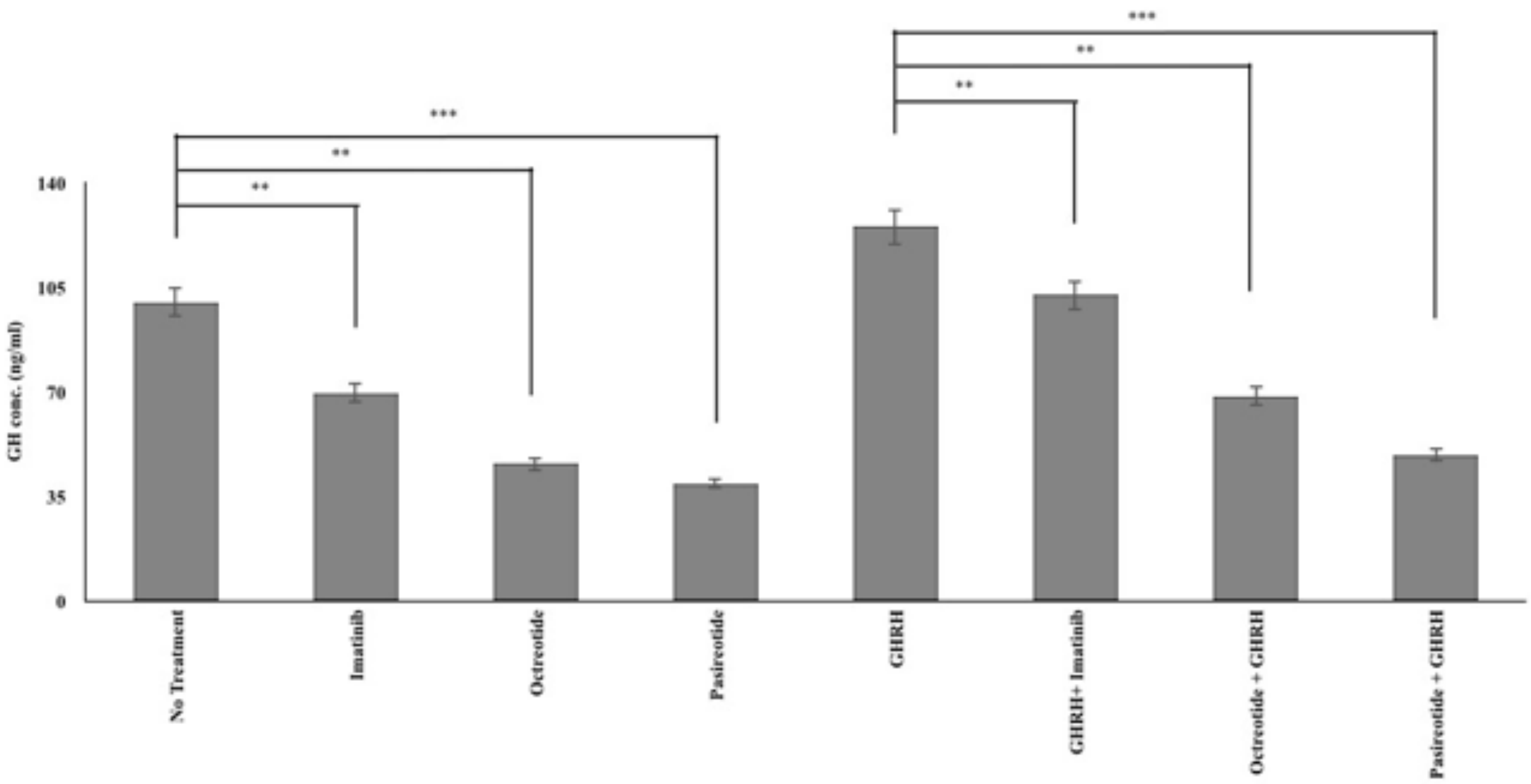

Figure 4: Graph showing effect of GH on cells treated with imatinib, octreotide, pasireotide and in combination with GHRH.

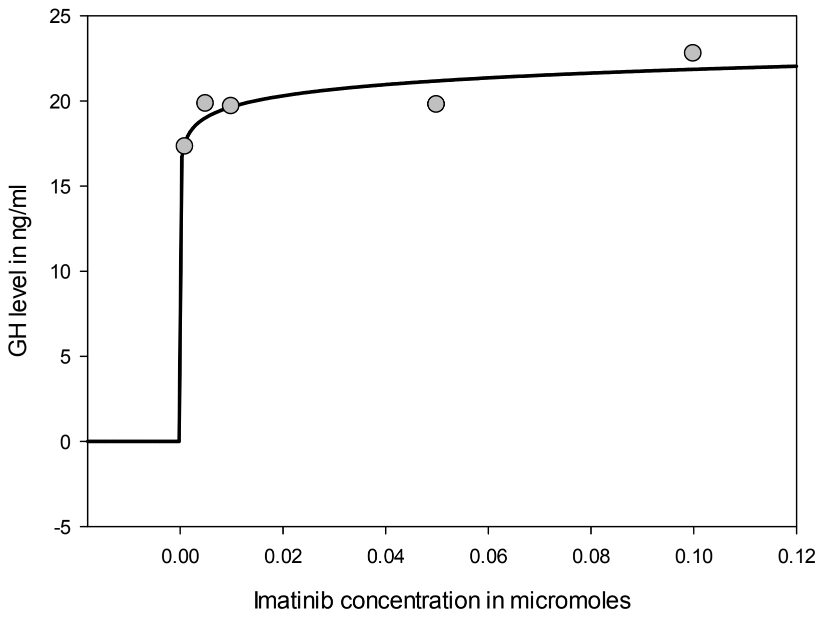

Figure 5: ED50 from imatinib treated cells. ED50 calculated from our experiment was 0.015 µM

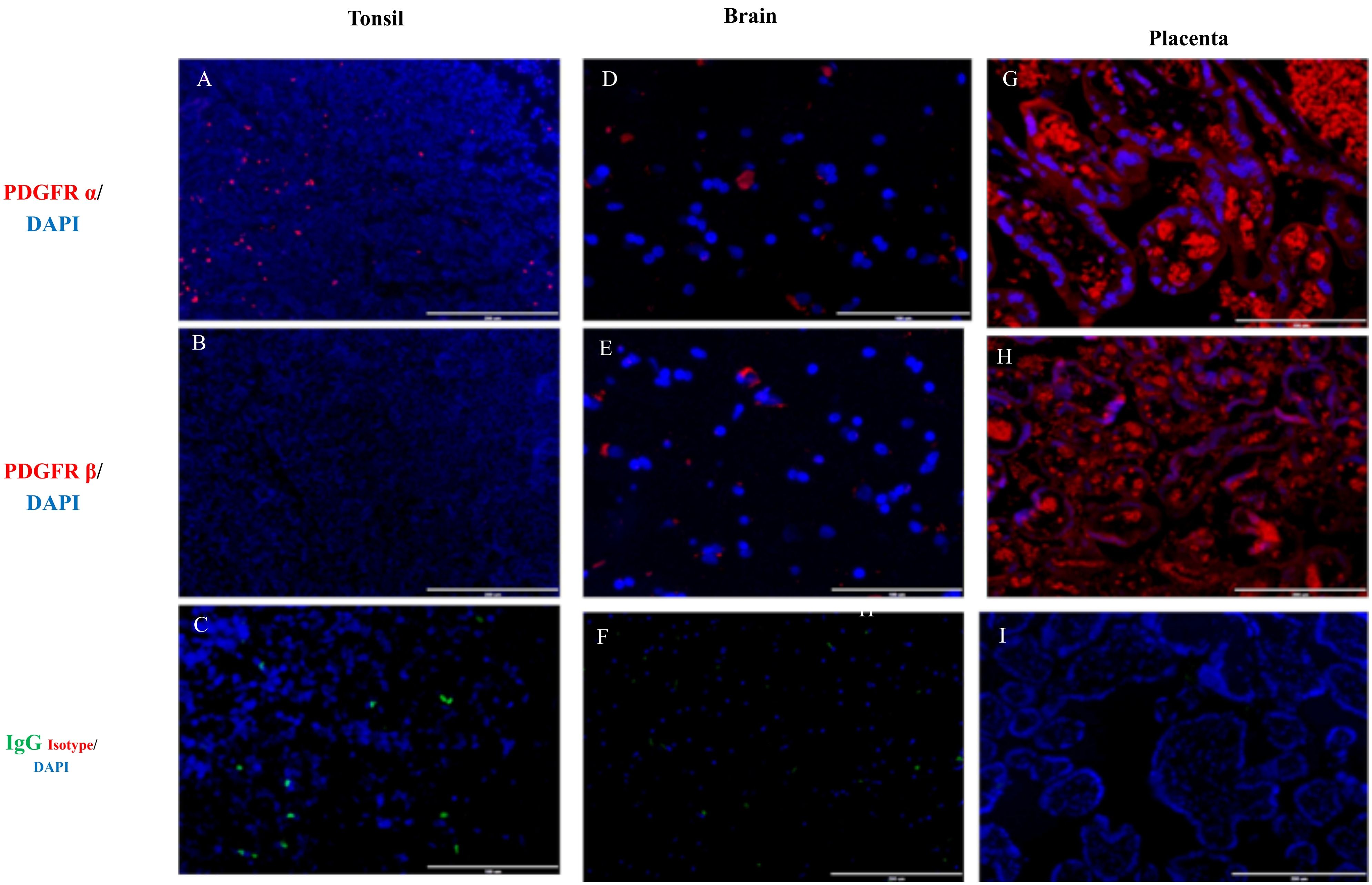

Figure 6: Controls for PDGFRα/β Immunoflouroscence. Immunoflouroscence for negative (Tonsil A-C and Brain D-F) and positive (Placenta,G-I)

tissue controls verifies the specificity of the PDGFRα/β antibodies. Significant expression of PDGFRα/β is seen in placenta. Also, control staining of the sections with an isotype-matched control (C,F,I) antibody without the primary antibody.
